# A retrospective study comparing definitive chemoradiotherapy vs. chemoradiotherapy followed by surgery in T4 esophageal squamous cell carcinoma patients who were downstaged after neochemoradiotherapy

**DOI:** 10.1186/s13014-022-02116-0

**Published:** 2022-08-23

**Authors:** Tian Zhang, Zhoubo Guo, Xi Chen, Jie Dong, Hongjing Jiang, Peng Tang, Ping Wang, Dong Qian, Wencheng Zhang, Qingsong Pang

**Affiliations:** 1 Key Laboratory of Cancer Prevention and Therapy, Department of Radiation Oncology, National Clinical Research Center for Cancer, Tianjin’s Clinical Research Center for Cancer, Tianjin Medical University Cancer Institute and Hospital, Huanhu West Road, Hexi District, Tianjin, China; 2 Key Laboratory of Cancer Prevention and Therapy, Department of Nutrition Therapy, National Clinical Research Center for Cancer, Tianjin’s Clinical Research Center for Cancer, Tianjin Medical University Cancer Institute and Hospital, Tianjin, China; 3 Key Laboratory of Cancer Prevention and Therapy, Department of Esophageal Cancer, National Clinical Research Center for Cancer, Tianjin’s Clinical Research Center for Cancer, Tianjin Medical University Cancer Institute and Hospital, Tianjin, China; 4 Division of Life Sciences and Medicine, Department of Radiation Oncology, The First Affiliated Hospital of USTC, University of Science and Technology of China, Hefei, Anhui China

**Keywords:** Esophageal squamous cell carcinoma, T4, Neo-chemoradiotherapy followed by surgery, Definitive chemoradiotherapy

## Abstract

**Background:**

The outcome of patients with T4 esophageal squamous cell carcinoma (ESCC) is extremely poor. Two distinct therapeutic options are currently available for T4 esophageal cancers: neochemoradiotherapy followed by surgery (CRT-S) and definitive chemoradiotherapy (D-CRT). This study aimed to investigate the clinicopathologic characteristics of T4 ESCC in Chinese patients and compare the survival between the two therapeutic options.

**Methods:**

We retrospectively analyzed 125 patients with clinically unresectable T4 ESCC in Tianjin Medical University Cancer Institute and Hospital from January 2010 to December 2020. Overall survival (OS), progression-free survival (PFS) and associated factors were analyzed.

**Results:**

A total of 106 of 125 T4 ESCC patients were downstaged of the tumor by neoadjuvant CRT. Among 106 patients, 32 patients underwent CRT-S, and 74 patients underwent D-CRT. Patients in the CRT-S group had a higher OS (20.4 months vs. un-reached median OS, *p* = 0.037) and PFS (8.6 months vs. 21.0 months, *p* = 0.008) than those in the D-CRT group. In multivariate analysis, treatment was an independent predictor of PFS. After propensity score matching (PSM), 50 patients (CRT-S = 25; D-CRT = 25) were matched. Among these 50 patients, patients in the CRT-S group had a higher OS (15.6 months vs. un-reached median OS, *p* = 0.025) and PFS (7.2 months vs. 18.8 months, *p* = 0.026) than those in the D-CRT group. In multivariate analysis, treatment was an independent predictor for PFS.

**Conclusion:**

We demonstrated that CRT-S was superior to D-CRT for T4 ESCC patients who were downstaged by neo-CRT with respect to longer OS and PFS. Randomized controlled trials involving large population samples are needed to define the standard treatment for T4 ESCC.

## Introduction

Esophageal squamous cell carcinoma (ESCC) is one of the leading causes of cancer-related mortality worldwide, with a high prevalence in Asia [1, 2]. The outcome of patients with T4 esophageal cancer, defined as a tumor that invades neighboring structures (e.g., aorta, trachea, bronchus, and lung), is extremely poor [3]. Despite advances in surgical treatment, these tumors are usually considered inoperable; however, surgery alone has not improved the prognosis of patients with T4 esophageal tumors [4, 5].

Two distinct therapeutic options are currently available for T4 esophageal cancers: chemoradiotherapy followed by surgery (CRT-S), which comprises esophagectomy following downstaging of the tumor by CRT, and definitive chemoradiotherapy (D-CRT), which is designed to avoid esophagectomy by using maximum doses of irradiation [4, 6]. Until now, D-CRT has been the standard alternative curative management for patients with locally advanced disease who are not eligible for or refuse surgery [7]. To our knowledge, there is little information on the differences in the clinical outcomes of patients with T4 ESCC who undergo D-CRT and those who receive CRT-S. In this study, we discuss these two treatment modalities.

## Methods

### Patients

We retrospectively enrolled patients with ESCC who had been treated with CRT from January 2010 to December 2020 at our center, including 8 cases from a single-arm, single-center, investigator-initiated, exploratory, phase II clinical study (ClinicalTrials.gov NCT04137679) at Tianjin Medical University Cancer Institute & Hospital in Tianjin, China. The inclusion criteria were as follows: (1) squamous cell histological type; (2) staged as unresectable T4 disease by a multidisciplinary team based on biopsy and imaging pretreatment examination data; (3) downstaged to T3 or lower T stage by neoadjuvant CRT; (4) without distant organ metastases; and (5) Eastern Cooperative Oncology Group (ECOG) performance status (PS) of ≤ 1. All patients were staged according to the 8th edition of the American Joint Committee on Cancer (AJCC) staging manual.

Adjacent organ invasion was diagnosed by esophageal endoscopy ultrasound (EUS), endobronchial ultrasonography (EBUS), bronchoscopy, and/or computed tomography (CT) according to the following criteria: 1) EUS showed invasion of the trachea, bronchus, aorta, pleura, pericardium, and/or other peripheral organs; 2) EBUS showed invasion of the trachea and/or bronchus; 3) bronchoscopy showed protrusion of the esophageal tumor into trachea and/or bronchi or abnormal tracheal mucosa; and 4) if the patient could not undergo EUS, adjacent organ invasion was defined by CT or positron emission tomography (PET)-CT. Generally, invasion of the peripheral organs was diagnosed based on the loss of fat planes between the esophagus and peripheral organs. Invasion of the aorta was defined as > 90 degrees of the aorta surrounded by tumor in more than one CT slice. Invasion of the pericardium was defined as the disappearance of the fat line at the focus level and local pericardial thickening.

The following clinicopathologic parameters for each patient were also collected: sex, age at diagnosis, smoking history, drinking history and TNM stage in line with the 8th edition of the esophageal cancer staging system. The PFS and OS of patients diagnosed from January 2010 to December 2020 were recorded based on a follow-up clinic visit or a telephone call.

### Treatment and response

Patients received conventional fraction radiotherapy using a 6 MeV linear accelerator. The radiation dose was calculated by intensity-modulated radiotherapy (IMRT). All plans were based on 3- or 5-mm CT scan images obtained in the treatment position before radiotherapy. The gross tumor volume (GTV) included the esophageal tumor found under CT and esophageal endoscopy and the enlarged locoregional lymph nodes found by CT before treatment. GTV also included distant lymph nodes with metastasis for patients with cervical or abdominal distant lymph node metastases. The clinical target volume (CTV) was added to the esophageal tumor 5–6 mm laterally and 2–3 cm in the cephalo–caudal direction, and CTV also includes positive lymph nodes and its drainage area. The planning target volume (PTV) was added to the CTV 5 mm in the cephalo–caudal and lateral directions. Cone-beam computed tomography (CBCT) was used to guarantee the treatment position during the whole radiotherapy process once a week. The induction and concurrent chemotherapy with radiation included cisplatin, 5-FU or taxane-based regimens. The patients’ response to treatment was assessed by CT scan, endoscopy and upper gastrointestinal contrast in accordance with Response Evaluation Criteria in Solid Tumors 1.1 (RECIST 1.1) criteria and the method reported in our previous study [8].

Patients in the CRT-S group received a 40-Gy radiation dose (2 Gy once daily in 20 fractions, 5 days a week) and were scheduled to undergo resection 4–6 weeks after having completed induction chemoradiation. Patients in the D-CRT group received a 60-Gy radiation dose (2 Gy once daily in 30 fractions, 5 days a week). An intensity-modulated radiotherapy dose planning system was used.

### Propensity score matching analysis

In this study, propensity score matching (PSM) was used to reduce bias due to an imbalance in observed variables between the CRT-S and D-CRT groups. Three baseline characteristics (sex, T-stage and segment) were selected as covariates in the PSM model, and the match tolerance was set to 0.01. Propensity scores of individuals were calculated with logistic regression analysis (SPSS version 22.0, Chicago, IL), and then, the optimal 1:1 matching between CRT-S and D-CRT patients was produced based on propensity scores. After matching, the distribution of the remaining observed variables was similar in the CRT-S and D-CRT groups.

### Statistical analysis

Pearson’s χ^2^ test was used to investigate the correlations between 2 categorical variables. PFS and OS distribution was analyzed using the Kaplan–Meier method, and log-rank tests were employed for comparison of PFS or OS between 2 categories in univariate analysis. Multivariate survival analysis was conducted using Cox proportional hazards regression to identify independent prognostic factors. Data were statistically analyzed using SPSS 22.0 (Abbott Laboratories, North Chicago, IL, USA). Statistical significance was set at *p* < 0.05.

### Ethics statement

This study was approved by the institutional review board at Tianjin Medical University. Written informed consent was obtained from each patient to allow their biological samples to be genetically analyzed. The experimental protocol of this study was performed strictly in accordance with the guidelines.

## Results

**Fig. 1:**
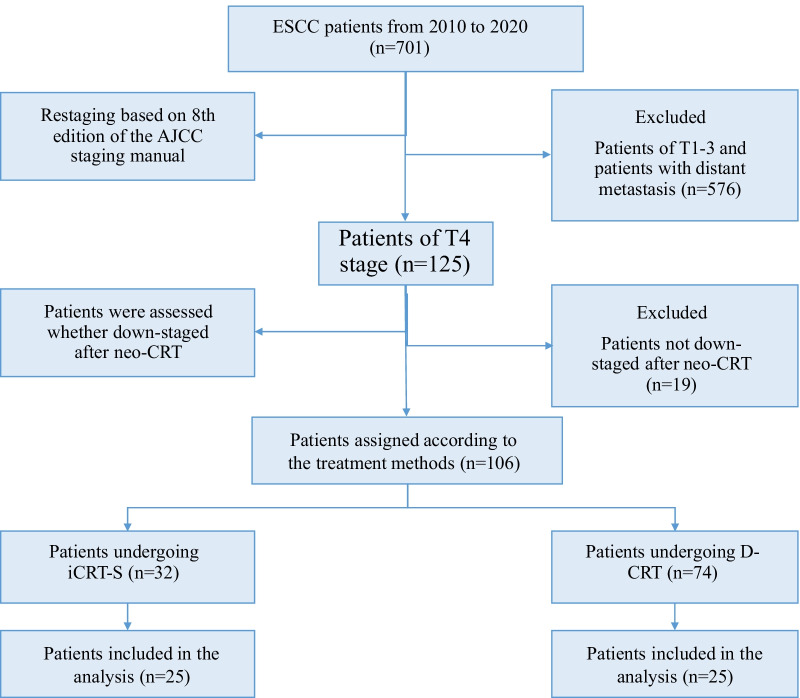
CONSORT diagram. Flow chart of patient inclusion into the study

From January 2010 to December 2020, 125 T4 ESCC patients were treated with CRT in our center; of these, 106 patients were down staged to T3 or lower T stage. The patients’ demographics and tumor characteristics are listed in Table 1. After multi-disciplinary treatment (MDT) of department of radiation oncology and department of esophageal cancer, all patients were suitable for surgery or definitive chemoradiotherapy after neochemoradiotherapy. Of the whole 106 cases, 8 cases were from a single-arm, single-center, investigator-initiated, exploratory, phase II clinical study (ClinicalTrials.gov NCT04137679) at Tianjin Medical University Cancer Institute & Hospital in Tianjin, China. The choices of the 8 patients were randomly made according to the random system. For the rest of the 98 cases, doctors provided the two possibilities of treatment ways equally and patients and doctors made the final decision together. Of the 106 downstaged T4 stage patients, 32 patients received surgery after neo-CRT, and 74 patients received D-CRT (Fig. 1). These patients had a median follow-up duration of 17.3 months (range: 2.6–90.6 months). The cohort consisted mostly of males, 92 male patients (86.8%) and only 14 female patients (13.2%). The majority of enrolled patients had upper (36; 34.0%) or middle (42; 4.0%) thoracic ESCC. In comparison with the D-CRT group, the CRT-S group had a lower proportion of T4b stage (*p* = 0.063), and more patients were in the middle or lower segment (*p* < 0.001). No other significant differences were found between the patients in the CRT-S and D-CRT groups with respect to age, sex, smoking history, drinking index, Karnofsky Performance Status (KPS) score, N stage or weight loss (Table 1).

**Table 1:** Comparison of clinical characteristics between ESCCs undergoing CRT-S and D-CRT before and after PSM

Characteristics		Before PSM				After PSM			
		Total	CRT-S	D-CRT	*P*	Total	CRT-S	D-CRT	*P*
N. of patients		106	32	74		50	25	25	
Age, years	≤ 60	53	12	41	0.091	26	11	15	0.258
	> 60	53	20	33		24	14	10	
Sex	Male	92	30	62	0.164	46	23	23	1.000
	Famale	14	2	12		4	2	2	
KPS score	> 80	75	21	54	0.552	34	17	17	0.431
	≤ 80	23	5	18		11	4	7	
	Unknown	8	6	2		5	4	1	
Smoking status	Ever	76	26	51	0.191	12	6	6	1.000
	Never	29	6	23		38	19	19	
Smoking index	< 850	96	30	66	0.461	47	24	23	0.552
	≥ 850	10	2	8		3	1	2	
Drinking index	> 4500	38	13	25	0.714	22	9	13	0.295
	refere ≤ 4500	62	16	46		23	13	10	
	Unknown	6				5	3	2	
T stage	T4a	55	21	34	0.063	28	14	14	1.000
	T4b	51	11	40		22	11	11	
N stage	N0	15	3	12	0.423	4	0	4	0.210
	N1	43	13	30		21	11	10	
	N2	33	13	20		19	11	8	
	N3	15	3	12		6	3	3	
Segment	Cervical	13	1	12	< 0.001	2	1	1	1.000
	Upper	36	5	31		10	5	5	
	Middle	42	16	26		32	16	16	
	Lower	14	10	4		6	3	3	
Weight loss	Yes	42	15	27	0.315	18	12	6	0.077
	No	64	17	47		32	13	19	

*PSM* propensity score matching; *ESCC* esophageal squamous cell carcinoma; *CRT-S* chemoradiotherapy followed by surgery; *D-CRT* definitive chemoradiotherapy; *KPS* Karnofsky Performance Status

**Fig. 2:**
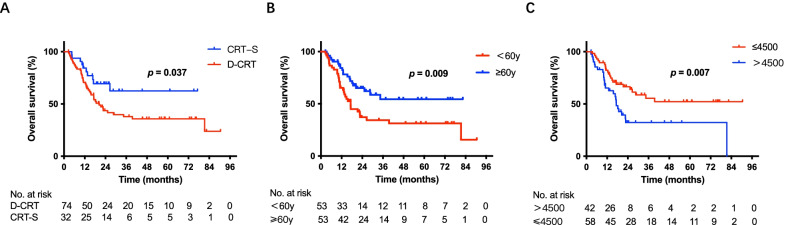
Overall survival (OS) of patients before PSM. **A** Kaplan–Meier survival curves for OS analyses of patients between the CRT-S and D-CRT groups. **B** Kaplan–Meier survival curves for OS analyses of patients of different age groups. **C** Kaplan–Meier survival curves for OS analyses of patients of different drinking index groups. OS: overall survival; PSM: propensity score matching; CRT-S: chemoradiotherapy followed by surgery; D-CRT: definitive chemoradiotherapy

**Fig. 3:**
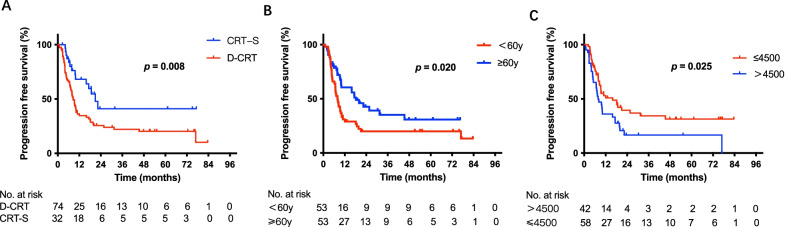
Progression-free survival (PFS) of patients before PSM. **A** Kaplan–Meier survival curves for PFS analyses of patients between the CRT-S and D-CRT groups. **B** Kaplan–Meier survival curves for PFS analyses of patients of different age groups. **C** Kaplan–Meier survival curves for PFS analyses of patients in different drinking index groups. PFS: progression free survival; PSM: propensity score matching; CRT-S: chemoradiotherapy followed by surgery; D-CRT: definitive chemoradiotherapy

Among all 106 patients, treatment (*p* = 0.037, Fig. 2A), age (*p* = 0.009, Fig. 2B), and drinking index (*p* = 0.007, Fig. 2C) were significantly associated with overall survival (OS); treatment (*p* = 0.008, Fig. 3A), age (*p* = 0.020, Fig. 3B), and drinking index (*p* = 0.025, Fig. 3C) were significantly correlated with progression-free survival (PFS). In multivariate analysis incorporating treatment, age and drinking index, drinking index (hazard ratio = 0.473, 95% confidence interval: 0.268–0.836, *p* = 0.010) was an independent predictor for OS. In multivariate analysis incorporating treatment, age and drinking index, treatment (hazard ratio = 0.459, 95% confidence interval: 0.242–0.869, *p* = 0.017) and drinking index (hazard ratio = 0.569, 95% confidence interval: 0.351–0.924, *p* = 0.022) were independent predictors for PFS (Tables 2 and 3).

**Table 2:** Univariate and multivariate analysis for OS before and after PSM

Characteristics		Before PSM				After PSM			
		Univariate analysis		Multivariate analysis		Univariate analysis		Multivariate analysis	
		*P*	HR (95% CI)	*P*	HR (95% CI)	*P*	HR (95% CI)	*P*	HR (95% CI)
Treatment	CRT-S	0.037	0.487	0.065	0.477	0.025	0.416	0.059	0.411
	D-CRT		0.244–0.972		0.214–1.046		0.188–0.921		0.163–1.033
Age, years	≤ 60	0.009	0.485	0.101	0.606	0.001	0.280	0.061	0.417
	> 60		0.278–0.848		0.332–1.103		0.125–0.629		0.167–1.041
Sex	Male	0.417	0.704			0.238	0.321		
	Famale		0.300–1.650				0.044–2.364		
KPS score	> 80	0.146	0.599			0.039	0.334	0.042	0.310
	≤ 80		0.297–1.206				0.113–0.992		0.100–0.960
Smoking status	Ever	0.681	1.137			0.092	2.420		
	Never		0.617–2.095				0.837–6.998		
Smoking index	< 850	0.203	1.667			0.039	3.377	0.380	1.780
	≥ 850		0.751–3.700				0.984–11.587		0.491–6.453
Drinking index	> 4500	0.007	0.464	0.010	0.473	0.072	0.485		
	≤ 4500		0.263–0.819		0.268–0.836		0.217–1.085		
T stage	T4a	0.642	1.138			0.211	1.599		
	T4b		0.660–1.961				0.761–3.363		
N stage	N0 N1 N2 N3	0.334	1.251 0.925–1.691			0.431	1.164 0.710–1.909		
Segment	Cervical Upper Middle Lower	0.959	0.929 0.677–1.275			0.177	0.903 0.476–1.711		
Weight loss	Yes	0.487	1.217			0.490	0.750		
	No		0.698–2.122				0.330–1.705		

*PSM* propensity score matching; *CRT-S* chemoradiotherapy followed by surgery; *D-CRT* definitive chemoradiotherapy; *KPS* Karnofsky Performance Status.*HR* hazard radio; *CI* confidence interval

**Table 3:** Univariate and multivariate analysis for PFS before and after PSM

Characteristics		Before PSM				After PSM			
		Univariate analysis		Multivariate analysis		Univariate analysis		Multivariate analysis	
		*P*	HR (95% CI)	*P*	HR (95% CI)	*P*	HR (95% CI)	*P*	HR (95% CI)
Treatment	CRT-S	0.008	0.463	0.017	0.459	0.026	0.463	0.049	0.488
	D-CRT		0.258–0.831		0.242–0.869		0.231–0.929		0.238–0.997
Age, years	≤ 60	0.020	0.570	0.119	0.671	0.001	0.312	0.009	0.372
	> 60		0.354–0.920		0.406–1.108		0.151–0.642		0.176–0.785
Sex	Male	0.142	0.561			0.121	0.236		
	Famale		0.257–1.227				0.032–1.727		
KPS score	> 80	0.149	0.647			0.056	0.422		
	≤ 80		0.357–1.174				0.170–1.050		
Smoking status	Ever	0.088	1.603			0.036	2.664	0.034	2.864
	Never		0.927–2.771				1.027–6.914		1.081–7.589
Smoking index	< 850	0.056	1.956			0.016	4.079	0.224	2.203
	≥ 850		0.969–3.948				1.174–14.169		0.616–7.881
Drinking index	> 4500	0.025	0.579	0.022	0.569	0.113	0.565		
	≤ 4500		0.357–0.938		0.351–0.924		0.276–1.157		
T stage	T4a	0.169	1.386			0.117	1.172		
	T4b		0.868–2.215				0.866–3.385		
N stage	N0 N1 N2 N3	0.224	1.197 0.913–1.568			0.323	1.014 0.644–1.594		
Segment	Cervical Upper Middle Lower	0.815	0.885 0.672–1.164			0.427	1.022 0.581–1.798		
Weight loss	Yes	0.757	0.927			0.800	0.913		
	No		0.575–1.495				0.450–1.852		

*PSM* propensity score matching; *CRT-S* chemoradiotherapy followed by surgery; *D-CRT* definitive chemoradiotherapy; *KPS* Karnofsky Performance Status.*HR* hazard radio; *CI* confidence interval

**Fig. 4:**
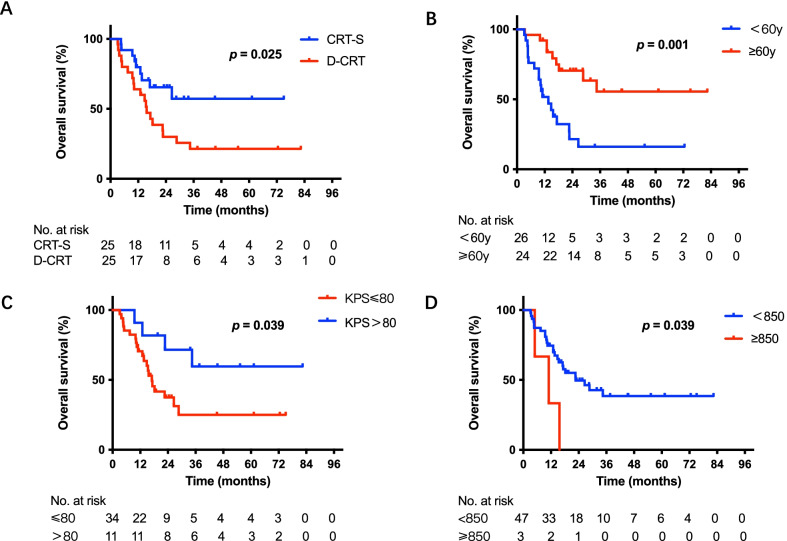
Overall survival (OS) of patients after PSM. **A** Kaplan–Meier survival curves for OS analyses for patients between the CRT-S and D-CRT groups. **B** Kaplan–Meier survival curves for OS analyses of patients of different age groups. **C** Kaplan–Meier survival curves for OS analyses of patients of different KPS groups. **D** Kaplan–Meier survival curves for OS analyses of patients of different smoking index groups. OS: overall survival; PSM: propensity score matching; CRT-S: chemoradiotherapy followed by surgery; D-CRT: definitive chemoradiotherapy; KPS: Karnofsky Performance Status

**Fig. 5:**
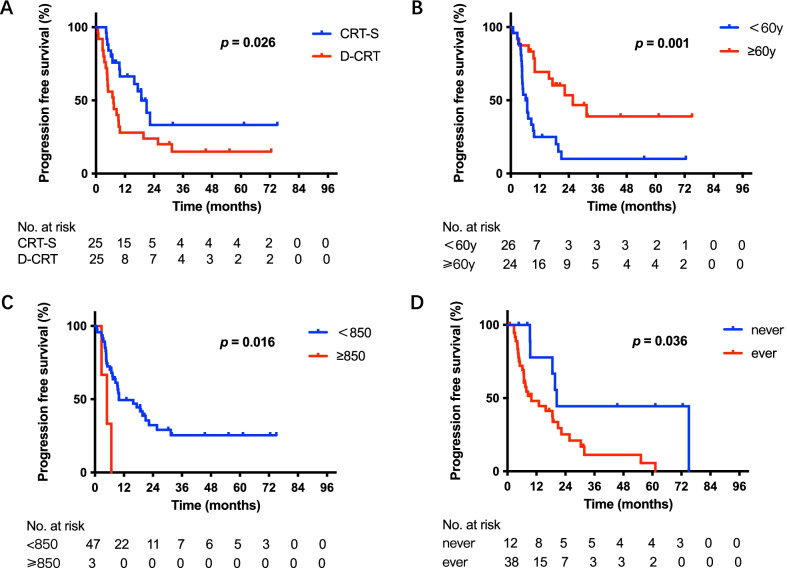
Progression-free survival (PFS) of patients after PSM. **A** Kaplan–Meier survival curves for PFS analyses of patients between the CRT-S and D-CRT groups. **B** Kaplan–Meier survival curves for PFS analyses of patients of different age groups. **C** Kaplan–Meier survival curves for PFS analyses of patients of different smoking index groups. **D** Kaplan–Meier survival curves for PFS analyses of patients of different smoking status groups. PFS: progression free survival; PSM: propensity score matching; CRT-S: chemoradiotherapy followed by surgery; D-CRT: definitive chemoradiotherapy

After propensity score matching (PSM), 50 patients (CRT-S = 25; D-CRT = 25) were matched, and Table 1 shows the patients’ characteristics. No significant differences between the CRT-S and D-CRT groups were observed in terms of either T stage or segment. Among these 50 patients, treatment (*p* = 0.025, Fig. 4A), age (*p* = 0.001, Fig. 4B), KPS score (*p* = 0.039, Fig. 4C) and smoking index (*p* = 0.039, Fig. 4D) were significantly associated with OS; treatment (*p* = 0.026, Fig. 5A), age (*p* = 0.001, Fig. 5B), smoking index (*p* = 0.016, Fig. 5C) and smoking status (*p* = 0.036, Fig. 5D) were significantly correlated with worse progression-free survival (PFS). In multivariate analysis incorporating treatment, age, KPS score, and smoking index, the KPS score (hazard ratio = 0.310, 95% confidence interval: 0.100–0.960, *p* = 0.042) was an independent predictor for OS. In multivariate analysis incorporating treatment, age, smoking status and smoking index, treatment (hazard ratio = 0.488, 95% confidence interval: 0.238–0.997, *p* = 0.049), age (hazard ratio = 0.372, 95% confidence interval: 0.176–0.785, *p* = 0.009) and smoking status (hazard ratio = 2.864, 95% confidence interval: 1.081–7.589, *p* = 0.034) were independent predictors for PFS (Tables 2 and 3).

For the D-CRT group, the complete response (CR) rate was 20.3% (15/74) and partial response rate (PR) rate was 79.7% (59/74). No additional treatment was given in the non-CR group until progression. For the CRT-S group, the CR rate was 12.5% (4/32), PR rate was 87.5% (28/32). The R0 resection rate was 93.75% (30/32) and R1 resection rate was 6.25% (2/32). The PCR rate was 37.5% (12/32) and non-PCR rate was 62.5% (20/32). No additional treatment was given in the non-R0 resected group after surgery until progression.

In the CRT-S group, most cases occurred with distant metastasis (8/32, 25.0%), and only one case occurred with local progression (1/32, 3.1%). In the D-CRT group, most cases had local progression (27/74, 36.5%), and the distant metastasis rate was 16.2% (12/74, *p* < 0.001, Table 4). In the CRT-S group, leukocytopenia incidence was 28.1%, neutrophilic granulopenia incidence was 18.8% and thrombocytopenia incidence was 12.5%. Three cases (9.4%) experienced anastomotic leakage, two cases occurred post thoracotomy pulmonary infection, and one of them died of complications after the operation. In the D-CRT group, leukocytopenia incidence was 41.4%, neutrophilic granulopenia incidence was 24.5%, and thrombocytopenia incidence was 28.7%. Six cases (6.4%) had esophageal fistula, and two cases (2.1%) had massive hemorrhage (Table 5).

**Table 4:** Type of first progression in CRT-S and D-CRT group

Type of first progression	CRT-S (n = 32)	D-CRT (n = 74)	*P*
Local recurrence	1	27	< 0.001
Regional lymph node metastasis	6	19	
Distant metastasis	8	12	
Stable	17	16	

*CRT-S* chemoradiotherapy followed by surgery; *D-CRT* definitive chemoradiotherapy

**Table 5:** Treatment-related adverse events

Adverse events		CRT-S, n (%)	D-CRT, n (%)	*P*
Anastomotic leakage		3 (9.4)	/	/
Post thoracotomy pulmonary infection		2 (6.3)	/	/
Operative mortality		1 (3.1)	/	/
Esophageal fistula		0 (0)	6 (6.4)	0.097
Massive hemorrhage		0 (0)	2 (2.1)	0.348
Radiation esophagitis	Grade 1	10 (31.3)	14 (14.9)	0.080
	Grade 2	10 (31.3)	13 (13.8)	
	Grade 3	0 (0)	5 (5.3)	
	Grade 4	0 (0)	0 (0)	
	Total	20 (62.5)	32 (34.0)	
Leukocytopenia	Grade 1	0 (0)	3 (3.2)	0.038
	Grade 2	9 (28.1)	26 (27.7)	
	Grade 3	0 (0)	10 (10.6)	
	Grade 4	0 (0)	0 (0)	
	Total	9 (28.1)	39 (41.4)	
Neutrophilic granulopenia	Grade 1	2 (6.3)	12 (12.8)	0.580
	Grade 2	3 (9.4)	6 (6.4)	
	Grade 3	1 (3.1)	4 (4.3)	
	Grade 4	0 (0)	1 (1.1)	
	Total	6 (18.8)	23 (24.5)	
Anemia	Grade 1	18 (56.3)	35 (34.0)	0.759
	Grade 2	3 (9.4)	10 (10.6)	
	Grade 3	0 (0)	1 (1.1)	
	Grade 4	0 (0)	0 (0)	
	Total	21 (65.6)	46 (48.9)	
Thrombocytopenia	Grade 1	3 (9.4)	20 (21.3)	0.087
	Grade 2	0 (0)	3 (3.2)	
	Grade 3	1 (3.1)	4 (4.3)	
	Grade 4	0 (0)	0 (0)	
	Total	4 (12.5)	27 (28.7)	
Hypoalbuminema	Grade 1	12 (37.5)	14 (14.9)	0.382
	Grade 2	0 (0)	2 (2.1)	
	Grade 3	0 (0)	0 (0)	
	Grade 4	0 (0)	0 (0)	
	Total	12 (37.5)	16 (17.0)	

*CRT-S* chemoradiotherapy followed by surgery; *D-CRT* definitive chemoradiotherapy

## Discussion

In the present study, we retrospectively investigated the clinical characteristics and prognosis of patients with T4 stage ESCC who were downstaged after neo-CRT. We found that patients in the CRT-S group had longer overall survival (OS, not available vs. 20.37 months) and progression-free survival (PFS, 21.0 vs. 8.6 months) than those in the D-CRT group. In addition, treatment option was an independent predictor for PFS. In the present report, the median OS of the CRT-S group was not available at the data cutoff (37.6 months median follow-up time). However, our findings showed that the 24-month OS rate was 69.4%.

The therapeutic strategy for locally advanced inoperable ESCC is controversial because the outcome differs among institutions [3, 6, 9–12]. For locally advanced inoperable ESCC, although concurrent chemoradiotherapy (CCRT) is recommended [13–15], neo-CRT is usually performed [10, 16]. Real-world data reported neoadjuvant chemoradiotherapy (44.9%) and definitive chemoradiotherapy (36.0%); however, 27.9% of patients undergoing neoadjuvant chemoradiotherapy did not receive planned esophagectomy [17]. In retrospective studies of T4b EC, patients who underwent surgical-based therapy had the longest median OS. However, the grouping was not random. Well-selected responders to chemotherapy (CMT) and/or radiotherapy (RT) may be able to undergo resection and numerically prolong survival, but patient selection remains paramount [18, 19]. The median OS in the CMT, RT and surgery-based therapy groups was 6.0, 12.7, and 43.9 months (*P* < 0.001), respectively. Nonsurgical treatment was associated with poorer OS (*P* < 0.05). A similar problem also existed in a study from Japan. A small sample study from Japan reported that the OS of T4b EC patients who underwent DCF-RT was 50% at 3 years compared to 37.5% for all T4b patients [18]. The mean interval was 17 months compared to 14.3 months for all included T4b patients. The 5-year survival rate was 19% in the D-CRT group [20, 21].

However, some studies showed negative results. A study from Japan enrolled 71 patients with T4 EC with tracheobronchial invasion (TBI); 58 underwent dCRT, and 13 underwent iCRT-S. There was no significant difference between the dCRT and iCRT-S groups. Clinical LN negativity and later treatment period were significantly good prognostic factors for T4 EC with TBI [10]. The median survival times (MSTs) of patients with locally advanced esophageal cancer with adjacent organ invasion after definitive CRT, bypass surgery plus CRT and CRT followed by esophagectomy were 10.4, 11.0 and 16.4 months, respectively; MST did not differ significantly between patients [22]. To our knowledge, our study is the first to compare CRT-S and D-CRT in locally advanced inoperable ESCC patients who were downstaged after neo-CRT. The choice of the 8 patients from a single-arm, single-center, investigator-initiated, exploratory, phase II clinical study (ClinicalTrials.gov NCT04137679) was randomly made according to the random system. For the rest of the 98 cases, doctors provided the two possibilities of treatment ways equally and patients and doctors made the final decision together. It is a basic principle to respect the patients right of consent. After propensity score matching (PSM), no significant differences between the CRT-S and D-CRT groups were observed in the patient characteristics. Although we acknowledge the limitations of retrospective studies, surgery after neo-CRT may probably improve survival and decrease recurrence compared with D-CRT for locally advanced inoperable ESCC patients who are downstaged after neo-CRT.

The first failure sites were categorized as local, regional nodal, or distant. In previous studies of locally advanced esophageal cancer patients who underwent chemoradiation followed by esophagectomy, the rates were 4.5–67% local, 10–57.9% regional nodal, and 21–32% distant failures [23–26]. Local failure was correlated with fewer lymph nodes (LNs) assessed and close/positive margins. Regional nodal failure was correlated with fewer LNs assessed and larger pretreatment tumor size. Distant recurrence was correlated with higher pathologic nodal stage, ulceration, perineural invasion, residual disease, and higher posttreatment PET SUV max. Patients with a pathologic complete response were less likely to experience distant recurrence [24]. In our study, all cases were evaluated as down staged to operable by CT, endoscopy and upper gastrointestinal contrast. Most cases in the CRT-S group had distant metastasis (8/32, 25.0%), and the local progression rate was 3.1% (1/32). The low local progression rate may be partially related to a high pathological complete response (pCR) rate (37.5%, 12/32), fewer lymph nodes (N0, 23/32; N1, 3/32; N2, 4/32; N3, 0/32), high negative margin rate (32/32, 100%) and high R0 resection rate (30/32, 93.75%) [24]. In the D-CRT group, the distant metastasis rate was only 16.2% (12/74). Most patients in the D-CRT group had local progression (27/74, 36.5%), 6 of whom were complicated with esophageal fistula, and 2 of whom had massive hemorrhage (Table 5). Our data indicated that CRT-S reduced the local recurrence rate and the incidence of esophageal fistula (*P* = 0.097) and massive hemorrhage (*p* = 0.348), which was partially consistent with previous studies [3, 27].

In a randomized study comparing D-CRT and CRT-S for patients with locally advanced operable esophageal carcinoma, preoperative chemoradiotherapy improved survival among patients with potentially curable esophageal or esophagogastric junction cancer. The regimen was associated with acceptable adverse event rates [28–30]. Some studies discussed the therapeutic options of T4b EC; although CRT-S improved the survival rate, perioperative complications were also increased. Anastomotic leakage after surgery was 25%, the recurrent laryngeal nerve paralysis rate was approximately 50% [21], the reoperation rate was approximately 7.7% [23], and the operative mortality was 5–23.4% [25]. In the present study, all adverse events were no more than grade 3 and manageable. Leukocytopenia incidence (*p* = 0.038) in the CRT-S group was lower than those in the D-CRT group. Although there were no differences of statistics in neutrophilic granulopenia incidence (*p* = 0.58), thrombocytopenia incidence (*p* = 0.087) and radiation esophagitis incidence (*p* = 0.080) in the CRT-S and D-CRT group, grade 3 radiation esophagitis incidence was lower in the CRT-S group than the D-CRT group. In the CRT-S group, anastomotic leakage, post thoracotomy pulmonary infection and operative mortality were lower than those in previous reports in patients with advanced ESCC (Table 5) [20, 21, 23, 25].

## Conclusions

In conclusion, the present study has demonstrated that CRT-S was superior to D-CRT for T4 ESCC patients who were downstaged by neo-CRT with respect to longer OS and PFS. However, the present study did have several limitations. First, this was a retrospective study from a single institution. Second, half of the patients included in the study were staged as T4 by CT imaging and not by endoscopy. Furthermore, the number of patients included in the study was small. Randomized controlled trials involving large population samples are needed to define the standard treatment for T4 esophageal cancer.

## Data Availability

Not appilicable.

## Abbreviations

ESCC: Esophageal squamous cell carcinoma
CRT-S: Neo-chemoradiotherapy followed by surgery
D-CRT: Definitive chemoradiotherapy
OS: Overall survival
PFS: Progression free survival
GTV: Gross tumor volume
CTV: Clinical target volume
PTV: Planning target volume
CBCT: Cone-beam computed tomography
ECOG: Eastern cooperative oncology group
PS: Performance status
AJCC: American joint committee on cancer
EUS: Esophageal endoscopy ultrasound
EBUS: Endobronchial ultrasonography
CT: Computed tomography
CMT: Chemotherapy
RT: Radiotherapy
CCRT: Concurrent chemoradiotherapy
PET: Positron emission tomography
IMRT: Intensity-modulated radiotherapy
RESIST: Response evaluation criteria in solid tumors
MDT: Multi-disciplinary treatment
TBI: Tracheobronchial invasion
PSM: Propensity score matching
PCR: Pathological complete response
KPS: Karnofsky Performance Status
CR: Complete response
PR: Partial response
LN: Lymph node
SUV: Standardized uptake value

## References

[1] Siegel RL, Miller KD, Jemal A (2019). Cancer statistics, 2019. CA Cancer J Clin.

[2] Liang H, Fan JH, Qiao YL (2017). Epidemiology, etiology, and prevention of esophageal squamous cell carcinoma in China. Cancer Biol Med.

[3] Makino T, Doki Y (2011). Treatment of T4 esophageal cancer: Definitive chemo-radiotherapy vs chemo-radiotherapy followed by surgery. Ann Thorac Cardiovasc Surg.

[4] Akutsu Y, Matsubara H (2015). Chemoradiotherapy and surgery for T4 esophageal cancer in Japan. Surg Today.

[5] Zhong H (2019). T4 esophageal cancer treated with radical resection. Panminerva Med.

[6] Makino T (2019). Treatment and clinical outcome of clinical T4 esophageal cancer: a systematic review. Ann Gastroenterol Surg.

[7] Ajani JA (2019). Esophageal and esophagogastric junction cancers, version 2.2019, NCCN clinical practice guidelines in oncology. J Natl Compr Canc Netw.

[8] Qian D (2019). Tumor remission and tumor-infiltrating lymphocytes during chemoradiation therapy: predictive and prognostic markers in locally advanced esophageal squamous cell carcinoma. Int J Radiat Oncol Biol Phys.

[9] Stahl M (2005). Chemoradiation with and without surgery in patients with locally advanced squamous cell carcinoma of the esophagus. J Clin Oncol.

[10] Yamaguchi S (2018). Long-Term outcome of definitive chemoradiotherapy and induction chemoradiotherapy followed by surgery for T4 esophageal cancer with tracheobronchial Invasion. Ann Surg Oncol.

[11] Fujita H (2005). Prospective non-randomized trial comparing esophagectomy-followed-by-chemoradiotherapy versus chemoradiotherapy-followed-by-esophagectomy for T4 esophageal cancers. J Surg Oncol.

[12] Fujita H (2005). Esophagectomy: is it necessary after chemoradiotherapy for a locally advanced T4 esophageal cancer? Prospective nonrandomized trial comparing chemoradiotherapy with surgery versus without surgery. World J Surg.

[13] Lyu J (2018). Outcomes of concurrent chemoradiotherapy versus chemotherapy alone for stage IV esophageal squamous cell carcinoma: a retrospective controlled study. Radiat Oncol.

[14] Ohkura Y (2019). Prognostic factors and appropriate lymph node dissection in salvage esophagectomy for locally advanced T4 esophageal Cancer. Ann Surg Oncol.

[15] Matsuda S (2019). Definitive chemoradiotherapy with simultaneous integrated boost of radiotherapy dose for T4 esophageal cancer-will it stand for a standard treatment?. J Thorac Dis.

[16] Akiyama Y (2020). Safety of thoracoscopic esophagectomy after induction chemotherapy for locally advanced unresectable esophageal squamous cell carcinoma. Asian J Endosc Surg.

[17] Hsu PK (2021). Treatment patterns and outcomes in patients with esophageal cancer: an analysis of a multidisciplinary tumor board database. Ann Surg Oncol.

[18] Cushman TR (2019). Management of unresectable t4b esophageal cancer: practice patterns and outcomes from the national cancer data Base. Am J Clin Oncol.

[19] Nakamura T (2006). Chemoradiotherapy with and without esophagectomy for advanced esophageal cancer. Hepatogastroenterology.

[20] Anderegg MCJ (2020). Feasibility of extended chemoradiotherapy plus surgery for patients with cT4b esophageal carcinoma. Eur J Surg Oncol.

[21] Hashimoto M (2020). Induction chemoradiotherapy including docetaxel, cisplatin, and 5-fluorouracil for locally advanced esophageal cancer. Esophagus.

[22] Hamai Y (2013). Treatment outcomes and prognostic factors for thoracic esophageal cancer with clinical evidence of adjacent organ invasion. Anticancer Res.

[23] Morita M (2012). Clinical significance of chemoradiotherapy and surgical resection for cT4 esophageal cancer. Anticancer Res.

[24] Shaikh T (2016). Patterns and predictors of failure following tri-modality therapy for locally advanced esophageal cancer. Acta Oncol.

[25] Garg PK (2016). Preoperative therapy in locally advanced esophageal cancer. World J Gastroenterol.

[26] Sugimura K (2021). Long-term results of a randomized controlled trial comparing neoadjuvant Adriamycin, cisplatin, and 5-fluorouracil vs docetaxel, cisplatin, and 5-fluorouracil followed by surgery for esophageal cancer (OGSG1003). Ann Gastroenterol Surg.

[27] Mori K (2021). Esophageal cancer patients' survival after complete response to definitive chemoradiotherapy: a retrospective analysis. Esophagus.

[28] van Hagen P (2012). Preoperative chemoradiotherapy for esophageal or junctional cancer. N Engl J Med.

[29] Kim JH (2001). Preoperative hyperfractionated radiotherapy with concurrent chemotherapy in resectable esophageal cancer. Int J Radiat Oncol Biol Phys.

[30] Zhang ZX (1998). Randomized clinical trial on the combination of preoperative irradiation and surgery in the treatment of adenocarcinoma of gastric cardia (AGC)–report on 370 patients. Int J Radiat Oncol Biol Phys.

